# Supplementary material for: Mapping the evolution of fertility support policies in China: A content and instrumental analysis
Source: PLoS One. 2025 Oct 9;20(10):e0332137. doi: 10.1371/journal.pone.0332137 (PMC12510515; doi:10.1371/journal.pone.0332137)
Supplement: S1 Appendix — (ZIP) [file pone.0332137.s001.zip › S1 Appendix. 226 original policy documents/94-劳动和社会保障部办公厅关于进一步加强生育保险工作的指导意见(FBM-CLI-4-55777).doc]

劳动和社会保障部办公厅关于进一步加强生育保险工作的指导意见

**发布部门：** [劳动和社会保障部(含劳动部)(已撤销)](https://www.pkulaw.com/law/chl?Aggs.IssueDepartment=60212) [机构沿革](javascript:void(0))

**发文字号：**劳社厅发〔2004〕14号

**发布日期：**2004.09.08

**实施日期：**2004.09.08

**时效性：** [现行有效](https://www.pkulaw.com/law/chl?Aggs.TimelinessDic=01)

**效力级别：** [部门规范性文件](https://www.pkulaw.com/law/chl?Aggs.EffectivenessDic=XE0302)

**法规类别：** [工资福利与劳动保险](https://www.pkulaw.com/law/chl?Aggs.Category=08707)

劳动和社会保障部办公厅关于
进一步加强生育保险工作的指导意见
（劳社厅发[2004]14号）

各省、自治区、直辖市劳动和社会保障厅(局)：
　　近十年来，各级劳动保障部门认真贯彻落实《企业职工生育保险试行办法》(劳部发[1994]504号)，生育保险工作取得了积极进展。为贯彻落实党的十六届三中全会精神，推进生育保险制度建设，加强生育保险管理，保障生育职工合法权益，现就进一步加强生育保险工作提出如下意见：
　　**一、**高度重视生育保险工作
　　建立生育保险制度，是我国社会主义市场经济发展和全面建设小康社会的必然要求，对促进经济和社会协调发展、保障妇女平等就业、促进企业公平竞争、维护妇女合法权益等方面具有重要作用。各级劳动保障部门要将建立和完善生育保险制度作为完善社会保障体系的一项重要任务，纳入当地劳动保障事业发展规划，逐步建立和完善与本地区经济发展相适应的生育保险制度。没有出台生育保险办法的地区，要积极创造条件，尽快建立生育保险制度。已经出台生育保险办法的地区，要逐步完善政策措施，确保生育保险制度稳健运行和可持续发展。

　　**二、**协同推进生育保险与医疗保险工作
　　各地要充分利用医疗保险的工作基础，以生育津贴社会化发放和生育医疗费用实行社会统筹为目标，加快推进生育保险制度建设。要充分利用医疗保险的医疗服务管理措施和手段，积极探索与医疗保险统一管理的生育保险医疗服务管理模式。各地要按照《[中国妇女发展纲要(2001—2010年)](https://www.pkulaw.com/chl/37733dd7246afab4bdfb.html)》提出的2010年城镇职工生育保险覆盖面达到90％的目标要求，制定发展规划，积极扩大参保范围。

　　**三、**切实保障生育职工的医疗需求和基本生活待遇
　　各地要按照国务院《[女职工劳动保护规定](https://www.pkulaw.com/chl/133408887a349cdabdfb.html)》(国务院令第9号)明确的产假期限和当地职工工资水平，合理确定生育津贴标准并及时支付，逐步实现直接向生育职工发放生育津贴，保障女职工生育期间的基本生活。暂不具备条件的地区，可以先实行生育医疗费用社会统筹，生育津贴由用人单位负担的办法，以保障生育职工的合法权益。生育保险筹资水平按照以支定收、收支基本平衡的原则合理确定，并及时调整。

　　**四、**加强生育保险的医疗服务管理
　　生育保险实行医疗机构协议管理，签订协议的医疗机构范围要考虑基本医疗保险定点医疗机构和妇产医院、妇幼保健院等医疗机构。社会保险经办机构在对这些医疗机构的保险管理、服务质量、信息管理等服务能力评价的基础上，选择适合生育保险要求的医疗机构签订生育保险医疗服务协议，明确双方的权利和义务。参保职工在生育保险协议医疗机构因生育所发生符合规定的医疗费用，由生育保险基金支付。生育保险医疗费用支付的范围原则上按照基本医疗保险药品目录、诊疗项目和医疗服务设施标准执行，具体支付办法由各地根据实际情况制定。
　　要积极探索生育医疗费用的结算办法，逐步实现社会保险经办机构与协议管理医疗机构直接结算。要加强对医疗服务费用的监督检查，控制不合理的支出，探索制定科学规范的生育医疗费用结算办法。在协议中明确监督检查措施和考核办法。要根据协议及时结算医疗费用，对不合理的医疗费用不予支付，对严重违反协议的医疗机构可以终止协议。采取向生育职工定额支付生育保险待遇的地区，应根据本地区职工工资水平、生育医疗费用实际支出等情况，合理确定待遇支付标准，并建立调整机制。

　　**五、**提高经办机构管理和服务水平
　　经办生育保险的社会保险经办机构要理顺管理职能，落实经费和人员，完善管理措施，加强基础建设，提高管理服务能力。要认真做好生育保险参保登记、保险费征缴和基金管理工作，加强医疗服务协议管理和生育保险津贴的社会化管理服务工作，简化经办流程，提高办事效率，为参保职工提供快捷、便利的服务。
　　　　　　　　　　　　　　　　　　　　　　　　　　　二00四年九月八日

© 北大法宝：（[www.pkulaw.com](https://www.pkulaw.com/)) 专业提供法律信息、法学知识和法律软件领域各类解决方案。北大法宝为您提供丰富的参考资料，正式引用法规条文时请与标准文本核对。
欢迎查看所有[产品和服务](https://www.pkulaw.com/helps/2.html)。法宝快讯：[如何快速找到您需要的检索结果？](https://www.pkulaw.com/helps/70.html)[法宝](https://www.pkulaw.com/helps/69.html)V6有何新特色？

扫描二维码阅读原文

**原文链接：**<https://www.pkulaw.com/chl/d66928dbab3a5ebebdfb.html>
